# Supplementary material for: Role of Silica on Clay-Catalyzed Ozonation for Total Mineralization of Bisphenol-A
Source: Molecules. 2023 Apr 30;28(9):3825. doi: 10.3390/molecules28093825 (PMC10179811; doi:10.3390/molecules28093825)
Supplement: Supplementary file 1 [file molecules-28-03825-s001.zip › molecules-2291552-supplementary.pdf]

Role of silica on clay-catalyzed ozonation for total mineralization of bisphenol-A

Farida Boudissa et al. A. Azzouz*

Supporting information

Table S1. physicochemical properties of BPA

Molecular formula	$C_{15}H_{16}O_2$
Structure	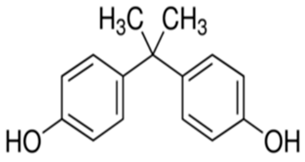
Molecular weight (g/mol)	228.29
Water solubility mg.L^{-1}(25 °C)	120–200
pKa	9.56–10.2
Ecotoxicology Log K_{ow}	3.32 at 25 °C

Calibration and absorptivity coefficient assessment

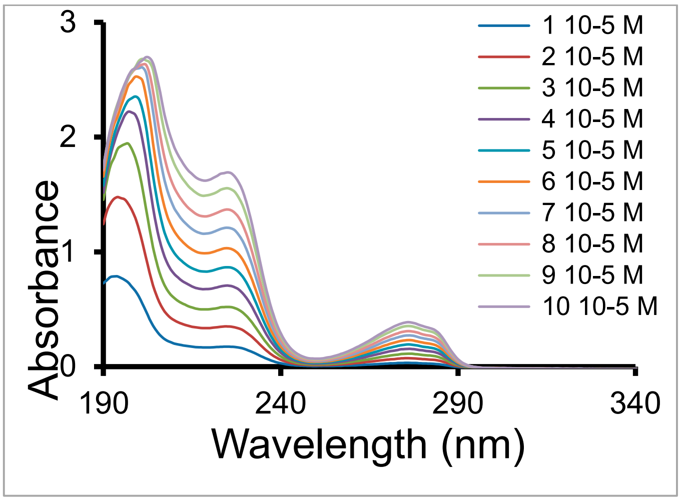

Figure S1. BPA UV-vis spectra at different concentrations in water. T= 22°C, pH=5.64, Quartz cell=1 cm.

Table S2. Molar extinction coefficient (ε) of the different UV-Vis bands

	Max wavelength (nm)	Molar extinction coefficient (ε) (M^{-1}.cm^{-1})*	Correlation coefficient R^2
	200	34530	0.6742
BPA	225	17170	0.9996
	278	3760	0.9993

* The coefficient were assessed from the slope of the linear part of the calibration curve fulfilling the Beer's law ($A=\varepsilon bc$), where A is the absorbance of BPA solution, ε is the molar absorptivity (M^{-1} cm^{-1}), b is the path length (cm), and c is the BPA concentration (M).

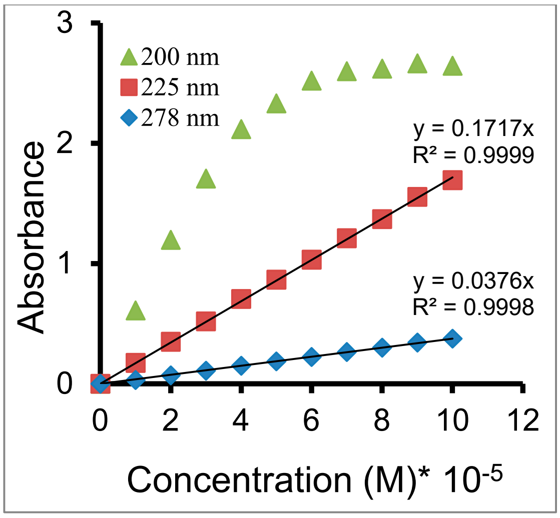

Figure S2. Calibration curves for BPA in water. T = 22 °C. pH = intrinsic value. Quartz cell = 1 cm.

Effect of montmorillonite addition

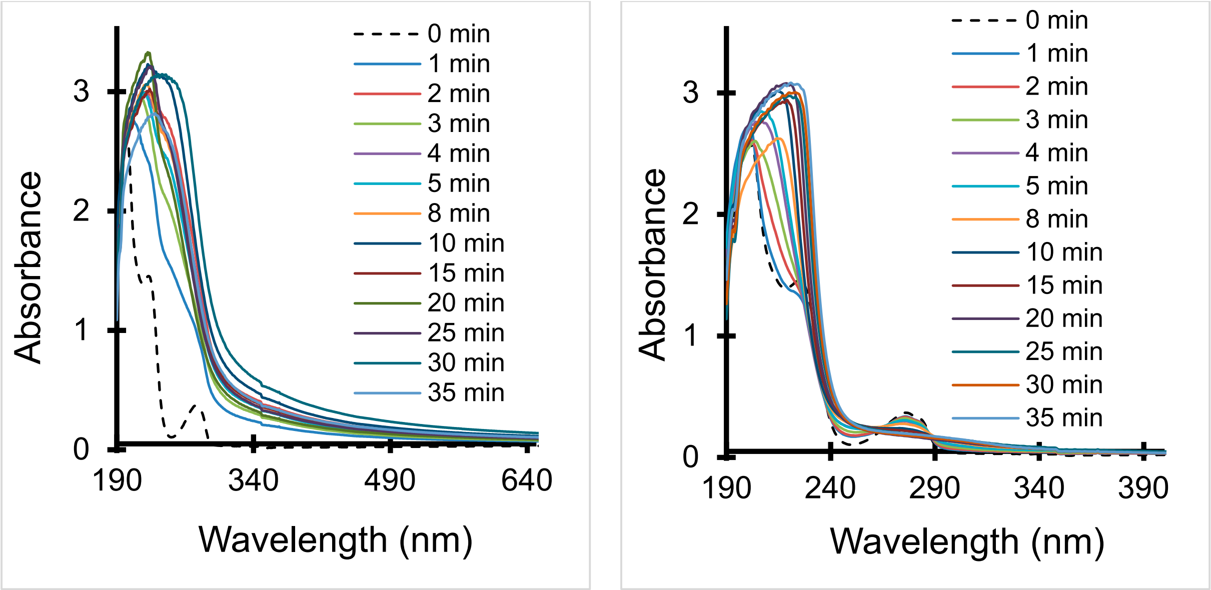

a **b**

Figure S3. Evolution in time of the UV-vis spectra of BPA during ozonation in presence of NaMt (***a***) and Fe(II)Mt (***b***). T = 22 °C. pH = intrinsic value. O_3 throughput: 600 mg.h^{-1}. Sample volume = 20 mL. Initial BPA concentration: 10^{-4} M. Catalyst concentration: 2 g.L^{-1}.

Effect of acid-activated bentonite addition

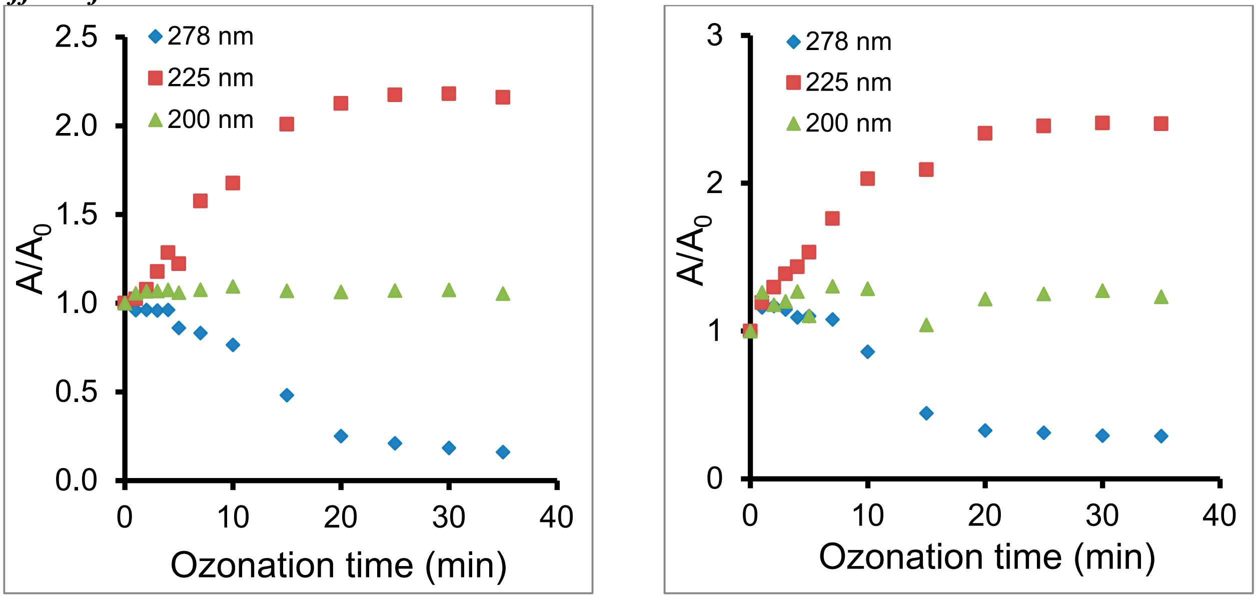

a. HMt-4 **b. HMt-15**

Figure S4. BPA relative absorbance of the 278 nm band during ozonation in distilled water. T = 22 °C. pH = intrinsic. Concentration: 10^{-4} M. Catalyst concentration: 2 g.L^{-1}.

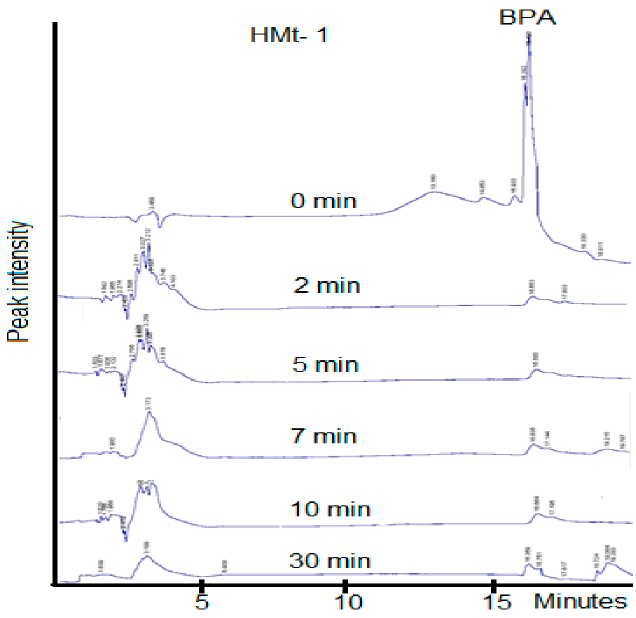

Figure S5. Evolution of the HPLC diagram of the reaction mixture during HMt-1-catalyzed ozonation (***a***) and of BPA peak as detected by UV-Vis at 278 nm (***b***). T = 22 °C. pH = intrinsic. BPA concentration: 10^{-4} M. Catalyst concentration: 2 g.L^{-1}. O_3 throughput: 600 mg.h^{-1}. Sample volume = 20 mL.

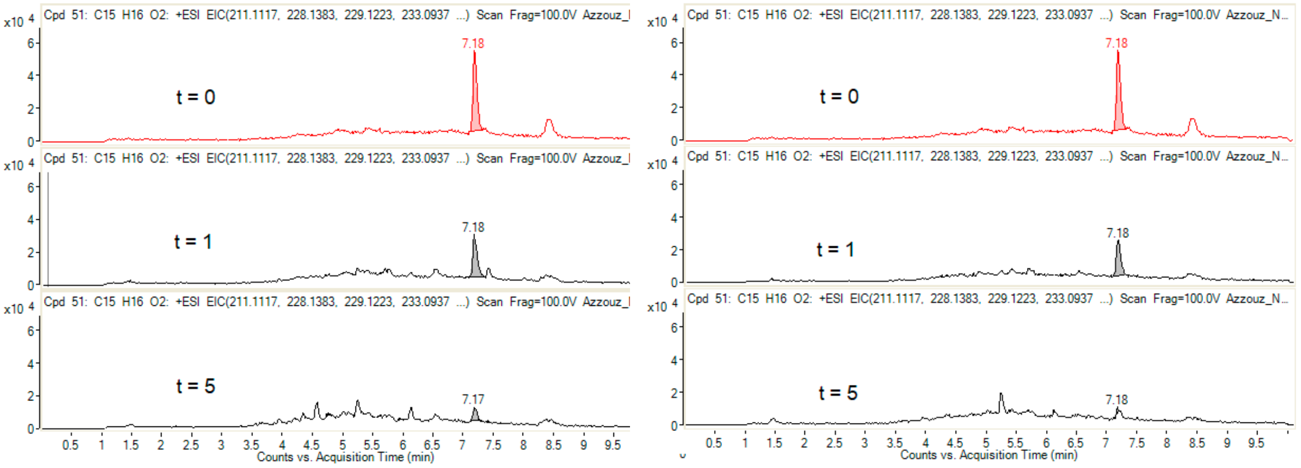

a **b**

Figure S6. LC-ToF-MS diagram of BPA ozonation mixture in the absence (a) and presence of Fe(II)Mt (b). Initial BPA concentration = 100 μ M.

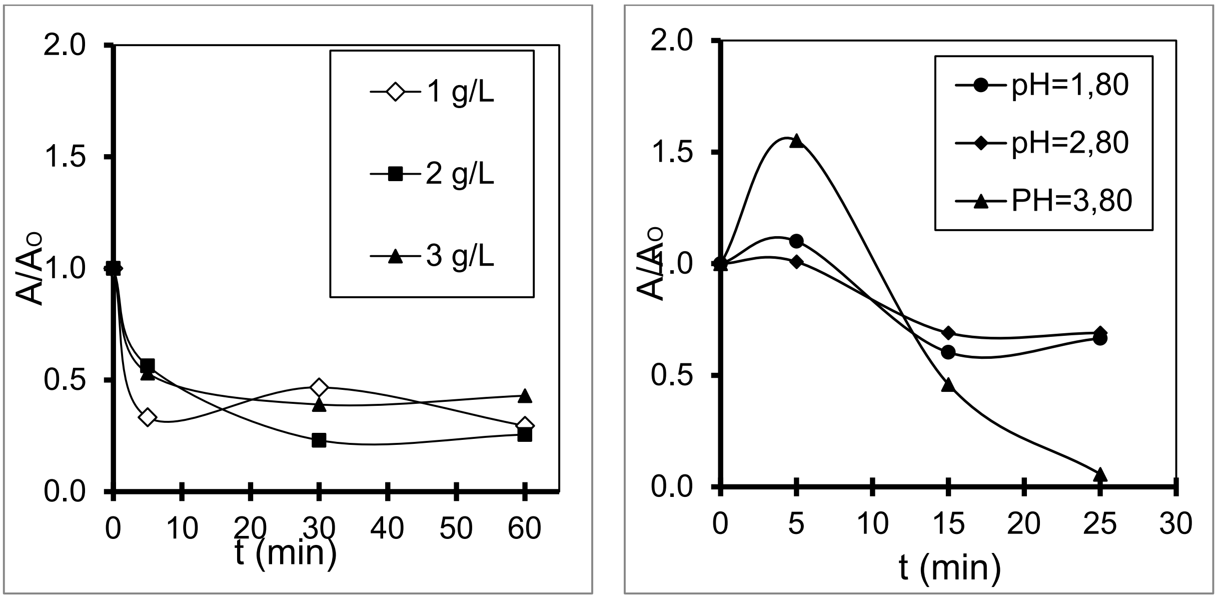

a **b**

Figure S7. Evolution in time of A/Ao of the 278 nm band during BPA adsorption on different amounts of hematite at intrinsic pH (***a***) and ozonation at different pH in the presence of 1 $g.L^{-1}$ of hematite (***b***). O_3 throughput: 600 $mg.h^{-1}$. Sample volume = 20 mL. Initial concentration: 10^{-4} M.
